# Supplementary material for: Mapping the evolution of fertility support policies in China: A content and instrumental analysis
Source: PLoS One. 2025 Oct 9;20(10):e0332137. doi: 10.1371/journal.pone.0332137 (PMC12510515; doi:10.1371/journal.pone.0332137)
Supplement: S1 Appendix — (ZIP) [file pone.0332137.s001.zip › S1 Appendix. 226 original policy documents/223-财政部、人口计生委关于印发《人口和计划生育事业单位财务制度》的通知(2012修订)(FBM-CLI-4-193183).doc]

财政部、人口计生委关于印发《人口和计划生育事业单位财务制度》的通知(2012修订)

**发布部门：** [财政部](https://www.pkulaw.com/law/chl?Aggs.IssueDepartment=60210) [国家人口和计划生育委员会(含国家计划生育委员会)(已撤销)](https://www.pkulaw.com/law/chl?Aggs.IssueDepartment=60304) [机构沿革](javascript:void(0))

**发文字号：**财教〔2012〕507号

**发布日期：**2012.12.28

**实施日期：**2013.01.01

**时效性：** [现行有效](https://www.pkulaw.com/law/chl?Aggs.TimelinessDic=01)

**效力级别：** [部门工作文件](https://www.pkulaw.com/law/chl?Aggs.EffectivenessDic=XE0303)

**法规类别：** [行政事业财务管理](https://www.pkulaw.com/law/chl?Aggs.Category=03305)

财政部、人口计生委关于印发《人口和计划生育事业单位财务制度》的通知
（财教〔2012〕507号）

各省、自治区、直辖市、计划单列市财政厅（局）、人口计生委，新疆生产建设兵团财务局、人口计生委：
　　为进一步规范人口和计划生育事业单位的财务行为，加强财务管理和监督，促进人口和计划生育事业的健康发展，根据《[事业单位财务规则](https://www.pkulaw.com/chl/f94d6af0dfbc1f85bdfb.html)》（财政部令第68号）和国家有关法律制度，结合人口和计划生育事业单位特点，我们对《计划生育事业单位财务制度》（财文字〔1997〕274号）进行了修订。现将修订后的《人口和计划生育事业单位财务制度》印发你们，请遵照执行。执行中有何问题，请及时反馈我部。
　　附件：人口和计划生育事业单位财务制度

财政部
人口计生委
2012年12月28日

　　附件：

人口和计划生育事业单位财务制度

　第一章　总　则

　　**第一条**　为了进一步规范人口和计划生育事业单位的财务行为，加强人口和计划生育事业单位财务管理和监督，提高资金使用效益，保障人口和计划生育事业单位的健康发展和计划生育基本国策的贯彻落实，根据《[事业单位财务规则](https://www.pkulaw.com/chl/f94d6af0dfbc1f85bdfb.html)》，结合人口和计划生育事业单位的特点，制定本制度。

　　**第二条**　本制度适用于各级各类人口和计划生育事业单位（以下简称人口和计划生育事业单位）的财务活动。

　　**第三条**　人口和计划生育事业单位财务管理的基本原则是：执行国家有关法律、法规和财务规章制度；坚持勤俭办事业的方针；正确处理事业发展需要和资金供给的关系，社会效益和经济效益的关系，国家、单位和个人三者利益的关系。

　　**第四条**　人口和计划生育事业单位财务管理的主要任务是：合理编制单位预算，严格预算执行，完整编制单位决算，真实反映单位财务状况；依法组织收入，努力节约支出；建立健全财务管理制度，加强经济核算，建立科学的财务考核指标体系，实施绩效评价，提高资金使用效益；加强资产管理，合理配置、有效利用、规范处置资产，防止资产流失；加强对单位经济活动的财务控制和监督，定期进行财务分析，防范财务风险。

　　**第五条**　人口和计划生育事业单位应当按照国家有关规定设置财务会计机构，配备具备从业资格的财务会计人员；不具备条件的，可以实行会计委托代理记账。

　　**第六条**　人口和计划生育事业单位的全部财务活动在单位负责人领导下，由单位财务部门统一管理。

　第二章　单位预算管理

　　**第七条**　单位预算是指人口和计划生育事业单位根据事业发展计划、目标和任务编制的年度财务收支计划。
　　人口和计划生育事业单位预算由收入预算和支出预算组成。

　　**第八条**　国家对人口和计划生育事业单位实行核定收支、定额或者定项补助、超支不补、结转和结余按规定使用的预算管理办法。
　　定额或者定项补助标准根据国家有关政策和财力可能，结合人口和计划生育事业特点、事业发展目标和规划、单位收支和资产状况等确定。定额或者定项补助可以为零。
　　非财政补助收入大于支出较多的人口和计划生育事业单位，可以实行收入上缴办法。具体办法由财政部门会同主管部门制定。

　　**第九条**　预算编制原则：
　　（一）坚持合法合规原则。根据国家有关方针政策、法律法规以及人口和计划生育事业发展目标和计划编制单位预算。
　　（二）坚持完整性和统一性原则。人口和计划生育事业单位必须将全部财务收支在预算中予以反映，并按照国家预算表格和统一的口径、程序及计算依据编制单位预算。
　　（三）坚持以收定支、收支平衡原则。单位预算编制要量入为出，收支平衡，不得编制赤字预算。
　　（四）坚持统筹兼顾、保证重点的原则。既要考虑事业发展的需要，又要考虑国家财力的可能和单位的收入状况、资产状况，保证重点，兼顾一般。
　　（五）坚持厉行节约、注重绩效原则。挖掘内部潜力，努力增收节支，提高资金使用效益。

　　**第十条**　人口和计划生育事业单位参考以前年度预算执行情况，根据预算年度的收入增减因素和措施，以及以前年度结转和结余情况，测算编制收入预算；根据事业发展需要与财力可能，测算编制支出预算。

　　**第十一条**　人口和计划生育事业单位根据年度事业发展目标、计划和预算编制的规定，提出预算建议数，经主管部门审核汇总报财政部门。人口和计划生育事业单位根据财政部门下达的预算控制数编制预算，由主管部门审核汇总报财政部门，经法定程序审核批复后执行。预算报送时间按财政部门的规定执行。

　　**第十二条**　人口和计划生育事业单位应当严格执行批准的预算。预算执行中，国家对财政补助收入和财政专户管理资金的预算一般不予调整。
　　上级下达的事业计划有较大调整，或者根据国家有关政策增加或者减少支出，对预算执行影响较大时，事业单位应当报主管部门审核后报财政部门调整预算；财政补助收入和财政专户管理资金以外部分的预算需要调增或者调减的，由单位自行调整并报主管部门和财政部门备案。
　　收入预算调整后，相应调增或者调减支出预算。

　　**第十三条**　人口和计划生育事业单位决算是指事业单位根据预算执行结果编制的年度报告。

　　**第十四条**　人口和计划生育事业单位应当按照规定，编制年度决算，由主管部门审核汇总后报财政部门审批。对财政部门批复调整的事项，人口和计划生育事业单位应及时调整。

　　**第十五条**　人口和计划生育事业单位应当加强决算审核和分析，保证决算数据的真实、准确，规范决算管理工作。

　第三章　收入管理

　　**第十六条**　收入是指人口和计划生育事业单位为开展业务及其他活动依法取得的非偿还性资金。

　　**第十七条**　人口和计划生育事业单位收入包括：
　　（一）财政补助收入，即人口和计划生育事业单位从同级财政部门取得的各类财政拨款。
　　（二）事业收入，即人口和计划生育事业单位开展专业业务活动及其辅助活动取得的收入，其中：按照国家有关规定应当上缴国库或者财政专户的资金，不计入事业收入；从财政专户核拨的资金和经核准不上缴国库或者财政专户的资金，计入事业收入。
　　（三）上级补助收入，即人口和计划生育事业单位从主管部门和上级单位取得的非财政补助收入。
　　（四）附属单位上缴收入，即人口和计划生育事业单位附属独立核算单位按照有关规定上缴的收入。
　　（五）经营收入，即人口和计划生育事业单位在专业业务活动及其辅助活动之外开展非独立核算经营活动取得的收入。
　　（六）其他收入，即本条上述规定范围以外的各项收入，包括投资收益、利息收入、捐赠收入等。

　　**第十八条**　事业收入包括：
　　（一）技术服务收入，即人口和计划生育事业单位开展计划生育优生优育咨询指导、避孕节育手术、孕前优生健康检查、孕情环情监测、计划生育手术并发症诊治、生殖健康检查治疗等技术服务活动取得的收入。
　　（二）病残儿鉴定收入，即人口和计划生育事业单位对病残儿进行鉴定所取得的收入。
　　（三）培训收入，即人口和计划生育事业单位开展业务培训取得的收入。
　　（四）宣传品制作收入，即人口和计划生育事业单位制作人口计生图书、音像等各类宣传品取得的收入。
　　（五）其他事业收入，即人口和计划生育事业单位开展专业业务活动及其辅助活动所取得的除上述各项收入以外的收入。

　　**第十九条**　经营收入包括：
　　（一）销售收入，即人口和计划生育事业单位非独立核算部门销售商品取得的收入。
　　（二）经营服务收入，即人口和计划生育事业单位非独立核算部门对外提供经营服务取得的收入。
　　（三）租赁收入，即人口和计划生育事业单位出租房屋、场地和设备取得的收入。
　　（四）其他经营收入，即人口和计划生育事业单位在专业业务活动及其辅助活动之外取得的除上述各项收入以外的收入。

　　**第二十条**　收入管理的要求：
　　（一）人口和计划生育事业单位应当在国家政策允许的范围内，依法组织收入，坚持社会效益为主，同时注重经济效益。
　　（二）人口和计划生育事业单位应当严格执行国家批准的收费项目和收费标准，不得擅自设立收费项目，并建立健全收费管理制度。
　　（三）人口和计划生育事业单位各项收入要及时入账，防止流失。
　　（四）人口和计划生育事业单位的各项收入应当全部纳入单位预算，统一核算，统一管理。

　　**第二十一条**　人口和计划生育事业单位对按照规定上缴国库或者财政专户的资金，应当按照国库集中收缴的有关规定及时足额上缴，不得隐瞒、滞留、截留、挪用和坐支。

　第四章　支出管理

　　**第二十二条**　支出是人口和计划生育事业单位开展业务及其他活动发生的资金耗费和损失。

　　**第二十三条**　人口和计划生育事业单位支出包括：
　　（一）事业支出，即人口和计划生育事业单位开展专业业务活动及其辅助活动发生的基本支出和项目支出。基本支出，即人口和计划生育事业单位为了保障其正常运转、完成日常工作任务而发生的人员支出和公用支出。项目支出，即人口和计划生育事业单位为了完成特定工作任务和事业发展目标，在基本支出之外所发生的支出。
　　（二）经营支出，即人口和计划生育事业单位在专业业务活动及其辅助活动之外开展非独立核算经营活动发生的支出。
　　（三）对附属单位补助支出，即人口和计划生育事业单位用财政补助收入之外的收入对附属单位补助发生的支出。
　　（四）上缴上级支出，即人口和计划生育事业单位按照财政和主管部门的规定，上缴上级单位的支出。
　　（五）其他支出，即本条上述规定范围之外的支出，包括利息支出、捐赠支出等。

　　**第二十四条**　人口和计划生育事业单位的各项支出应当全部纳入单位预算，建立健全支出管理制度。

　　**第二十五条**　人口和计划生育事业单位应当严格执行国家财务规章制度规定的开支范围及开支标准；国家没有统一规定的，由人口和计划生育事业单位做出规定，报主管部门和财政部门备案。
　　人口和计划生育事业单位的规定违反法律制度和国家政策的，主管部门和财政部门应当责令改正。

　　**第二十六条**　人口和计划生育事业单位在开展非独立核算经营活动中，应当正确归集实际发生的各项费用数；不能归集的，应当按照规定的比例合理分摊。经营支出应当与经营收入配比。

　　**第二十七条**　人口和计划生育事业单位从财政部门和主管部门取得的有指定项目和用途的专项资金，应当专款专用，定期向财政部门和主管部门报送专项资金使用情况；项目完成后，应当报送专项资金支出决算和使用效果的书面报告，接受财政部门和主管部门的检查和验收。

　　**第二十八条**　为了加强支出管理，提高经济核算水平，具备条件的人口和计划生育事业单位，可以根据开展业务及其他活动的实际需要，实行内部成本核算办法。

　　**第二十九条**　实行内部成本核算办法的范围：非财政补助收入能够基本满足正常支出的人口和计划生育管理服务机构。

　　**第三十条**　实行内部成本核算办法的单位、单位内部实行成本核算的部门，其成本费用应当按照支出用途分别归集到单位事业支出和经营支出的相应科目中。

　　**第三十一条**　人口和计划生育事业单位的基本建设支出、对外投资支出以及国家规定不得列入成本费用的其他支出，不得计入成本费用。

　　**第三十二条**　人口和计划生育事业单位应当严格执行国库集中支付制度和政府采购制度等有关规定。

　　**第三十三条**　人口和计划生育事业单位应当加强支出的绩效管理，提高资金使用的有效性。

　　**第三十四条**　人口和计划生育事业单位应当依法加强各类票据管理，确保票据来源合法，内容真实，使用正确，不得使用虚假票据。票据经办部门和人员应当对票据的真实性、合法性负责。财务部门和人员应当加强票据审核，杜绝报销虚假发票。

　第五章　结转和结余管理

　　**第三十五条**　结转和结余是指人口和计划生育事业单位年度收入与支出相抵后的余额。
　　结转资金是指当年预算已执行但未完成，或者因故未执行，下一年度需要按照原用途继续使用的资金。结余资金是指当年预算工作目标已完成，或者因故终止，当年剩余的资金。
　　经营收支结转和结余应当单独反映。

　　**第三十六条**　财政拨款结转和结余的管理，应当按照同级财政部门的规定执行。

　　**第三十七条**　非财政拨款结转按照规定结转下一年度继续使用。非财政拨款结余可以按照国家有关规定提取职工福利基金，剩余部分作为事业基金用于弥补以后年度单位收支差额；国家另有规定的，从其规定。

　　**第三十八条**　人口和计划生育事业单位应当加强事业基金的管理，在编制年度预算时统筹安排，合理使用，支出不得超出基金规模。

　第六章　专用基金管理

　　**第三十九条**　专用基金是指人口和计划生育事业单位按照规定提取或设置的有专门用途的资金。专用基金管理应当遵循先提后用、收支平衡、专款专用的原则，支出不得超出基金规模。

　　**第四十条**　专用基金包括：
　　（一）修购基金，即按照事业收入和经营收入的一定比例提取，并按照规定在相应的购置和修缮科目中列支（各列50%），以及按照其他规定转入，用于人口和计划生育事业单位固定资产维修、购置的资金。事业收入和经营收入较少的事业单位可以不提取修购基金。
　　（二）职工福利基金，即按照非财政拨款结余的一定比例提取转入，用于单位职工的集体福利设施、集体福利待遇等的资金。
　　（三）其他基金，即按照其他有关规定提取和设置的专用基金。

　　**第四十一条**　各项基金的提取比例和管理办法，国家有统一规定的，按统一规定执行，没有统一规定的由主管部门会同同级财政部门确定。

　第七章　资产管理

　　**第四十二条**　资产是指人口和计划生育事业单位占有或者使用的能以货币计量的经济资源，包括各种财产、债权和其他权利。

　　**第四十三条**　人口和计划生育事业单位的资产包括流动资产、固定资产、在建工程、无形资产和对外投资等。

　　**第四十四条**　人口和计划生育事业单位应当建立健全单位资产管理制度，加强和规范资产配置、使用和处置管理，维护资产安全完整，保障事业健康发展。

　　**第四十五条**　人口和计划生育事业单位应当按照科学规范、从严控制、保障事业发展需要的原则合理配置资产。根据单位资产存量状况、人员编制和有关资产配置标准，编制资产购置计划，按照部门预算管理的有关要求列入年度部门预算，并履行相关政府采购规定。

　　**第四十六条**　流动资产是指可以在一年以内（含一年）变现或者耗用的资产，包括现金、各种存款、零余额账户用款额度、应收及预付款项和存货等。

　　**第四十七条**　人口和计划生育事业单位建立健全现金及各种存款的内部管理制度，应当由财务部门按照国家有关规定开设、使用和管理银行账户。

　　**第四十八条**　人口和计划生育事业单位对应收款项和预付款项要按时清理结算，不得长期挂账。对确实无法收回的预付款，要查明原因，分清责任，按照规定权限，报经主管部门或财政部门批准后核销。

　　**第四十九条**　存货是指人口和计划生育事业单位在开展业务及其他活动中为耗用而储存的资产，包括材料、燃料、包装物、避孕药具、低值易耗品等。
　　（一）人口和计划生育事业单位应当建立健全存货管理制度。单位资产管理部门应指定专人负责，严格收发手续，完善存货验收、进出库和保管制度，防止丢失、损坏、变质。
　　（二）人口和计划生育事业单位资产管理部门应当建立材料明细账，定期与财务部门的材料总账进行核对，做到账账相符、账实相符。
　　（三）人口和计划生育事业单位应当制定材料储备定额和主要材料消耗定额。
　　（四）人口和计划生育事业单位应当对存货进行定期或不定期的清查盘点，保证账实相符。存货盘盈、盘亏应及时处理。

　　**第五十条**　固定资产是指使用期限超过一年，单位价值在1000元以上（其中：专用设备单位价值在1500元以上），并在使用过程中基本保持原有物质形态的资产。单位价值虽未达到规定标准，耐用时间在一年以上的大批同类物资，作为固定资产管理。
　　固定资产一般分为六类：房屋及构筑物；专用设备；通用设备；文物和陈列品；图书、档案；家具、用具、装具及动植物。
　　人口和计划生育事业单位的固定资产明细目录由国务院人口和计划生育主管部门制定，报国务院财政部门备案。

　　**第五十一条**　人口和计划生育事业单位应当加强固定资产管理。
　　（一）建立健全固定资产管理制度，加强固定资产维护和保养，制定操作规程，建立技术档案和使用情况报告制度。
　　（二）购建和调入的固定资产，由人口和计划生育事业单位资产管理部门负责验收，单位财务部门参与验收。购进专用设备和新建的房屋及建筑物竣工时，应有专业技术人员参加验收。经验收后的固定资产要及时入账并交付使用。
　　（三）人口和计划生育事业单位固定资产报废和转让，以及单位价值或者批量价值在规定限额以上的资产的处置应当按照有关规定，报主管部门和财政部门批准。
　　（四）人口和计划生育事业单位应当定期或者不定期对固定资产清查盘点。年度终了前应当进行一次全面的清查盘点，做到账实相符。对于盘盈、盘亏的固定资产应当及时按照规定处理。

　　**第五十二条**　在建工程是指已经发生必要支出，但尚未达到交付使用状态的建设工程。
　　人口和计划生育事业单位对建设工程项目，应按照国家有关规定，单独建账、单独核算，严格控制工程成本，工程完工后应尽快办理工程结算和竣工财务决算，并及时办理资产交付手续。国家另有规定的，从其规定。

　　**第五十三条**　无形资产是指不具有实物形态而能为使用者提供某种权利的资产，包括专利权、商标权、著作权、土地使用权、非专利技术以及其他财产权利等。
　　人口和计划生育事业单位应当对本单位无形资产的评估确认、开发、保护、使用和转让加强管理。
　　人口和计划生育事业单位转让无形资产，应当按照有关规定进行资产评估，取得的收入按照国家有关规定处理。取得无形资产发生的支出，应当计入事业支出。

　　**第五十四条**　对外投资是人口和计划生育事业单位依法利用货币资金、实物、无形资产等方式向其他单位的投资。
　　（一）人口和计划生育事业单位应当严格控制对外投资。在保证单位正常运转和事业发展的前提下，按照国家有关规定可以对外投资的，应当履行相关审批程序。
　　事业单位不得使用财政拨款及其结余进行对外投资，不得从事股票、期货、基金、企业债券等投资，国家另有规定的除外。
　　（二）人口和计划生育事业单位以非货币性资产对外投资的，应当按照国家有关规定进行资产评估，合理确定资产价值。
　　（三）对外投资应当进行充分的技术和经济效益论证，保证资产的保值、增值。
　　（四）人口和计划生育事业单位应建立健全对投资企业和项目的管理制度、认真履行出资人职责，加强财务监督。

　　**第五十五条**　人口和计划生育事业单位资产处置应当遵循公开、公平、公正和竞争、择优的原则，严格履行相关审批程序。出租、出借资产，应当按照国家有关规定经主管部门审核同意后报同级财政部门审批。

　　**第五十六条**　人口和计划生育事业单位应当提高资产使用效率，按照国家有关规定实行资产共享、共用。

　第八章　负债管理

　　**第五十七条**　负债是指人口和计划生育事业单位所承担的能以货币计量，需要以资产或者劳务偿还的债务。

　　**第五十八条**　人口和计划生育事业单位负债包括借入款项、应付款项、暂存款项、应缴款项等。
　　应缴款项包括人口和计划生育事业单位收取的应当上缴国库或者财政专户的资金、应缴税费，以及其他按照国家有关规定应当上缴的款项。

　　**第五十九条**　人口和计划生育事业单位应对不同性质的负债分别管理，及时清理并按照规定办理结算，保证各项负债在规定期限内归还。

　　**第六十条**　人口和计划生育事业单位应当建立财务风险控制机制，规范和加强借入款项管理，严格执行审批程序，不得违反规定举借债务和提供担保。

　第九章　事业单位清算

　　**第六十一条**　人口和计划生育事业单位发生划转、撤销、合并、分立时，应当进行清算。

　　**第六十二条**　人口和计划生育事业单位清算，应当在主管部门和财政部门的监督指导下，对单位的财产、债权、债务等进行全面清理，编制资产负债表、财产目录和债权、债务清单，提出财产作价依据和债权、债务处理办法，做好资产的移交、接收、划转和管理工作，并妥善处理各项遗留问题。

　　**第六十三条**　人口和计划生育事业单位清算结束后，经主管部门审核并报国有资产管理部门和财政部门批准，其资产分别按下列办法处理：
　　（一）因隶属关系改变，成建制划转的人口和计划生育事业单位，其全部资产无偿移交，并相应划转经费指标。
　　（二）转为企业管理的人口和计划生育事业单位，全部资产扣除负债后，转作国家资本金。需要进行资产评估的，按照国家有关规定执行。
　　（三）撤销的人口和计划生育事业单位，全部资产由主管部门和财政部门核准处理。
　　（四）合并的人口和计划生育事业单位，全部资产移交接收单位或新组建单位。合并后多余的国有资产，由主管部门和财政部门核准后处理。
　　（五）分立的人口和计划生育事业单位，资产按照有关规定移交分立后的人口和计划生育事业单位，并相应划转经费指标。

　第十章　财务报告与分析

　　**第六十四条**　财务报告是反映人口和计划生育事业单位一定时期财务状况和事业成果的总结性书面文件。
　　人口和计划生育事业单位应当按照财政部门和主管部门的规定和要求，定期向主管部门和财政部门以及其他有关的报表使用者提供财务报告。

　　**第六十五条**　人口和计划生育事业单位报送的年度财务报告包括资产负债表、收入支出表、财政拨款收入支出表、固定资产投资决算报表等主表，有关附表以及财务情况说明书等。

　　**第六十六条**　财务情况说明书，主要说明人口和计划生育事业单位收入及其支出、结转、结余及其分配、资产负债变动、对外投资、资产出租出借、资产处置、固定资产投资、绩效考评的情况，对本期或者下期财务状况发生重大影响的事项，以及需要说明的其他事项。

　　**第六十七条**　人口和计划生育事业单位财务分析的内容包括预算编制与执行、资产使用、负债情况、收入支出状况等。

　　**第六十八条**　财务分析指标分为财务指标和业务指标两类。
　　（一）财务指标包括：预算收入和预算支出完成率、人员支出与公用支出分别占事业支出的比率、人均基本支出、资产负债率、财政补助收入占总收入的比率、事业收入占总收入的比率、经营收入占总收入的比率、财政补助收入增长率、事业收入增长率、经营收入增长率、避孕药具库存量及库存结构等。
　　（二）业务指标包括：宣传品制作种类、宣传品发行量、宣传品进村入户率、人口和计划生育培训人次、避孕药具使用率、药具使用有效率、免费孕前检查覆盖率及技术服务总量等。
　　除上述指标外，人口和计划生育事业单位可以根据本单位业务特点增加财务分析指标。

　第十一章　财务监督

　　**第六十九条**　人口和计划生育事业单位财务监督主要包括对预算管理、收入管理、支出管理、结转和结余管理、专用基金管理、资产管理、负债管理等的监督。

　　**第七十条**　事业单位财务监督应当实行事前监督、事中监督、事后监督相结合，日常监督与专项监督相结合。

　　**第七十一条**　人口和计划生育事业单位应当建立健全内部控制制度、经济责任制度、财务信息披露制度等监督制度，按照规定公开财务信息。

　　**第七十二条**　人口和计划生育事业单位应当依法接受主管部门和财政、审计部门的监督。

　第十二章　附　则

　　**第七十三条**　人口和计划生育事业单位基本建设投资的财务管理，应当执行本制度，但国家基本建设投资财务管理制度另有规定的，从其规定。

　　**第七十四条**　参照[公务员法](https://www.pkulaw.com/chl/823ca97b7f55d18cbdfb.html)管理的人口和计划生育事业单位财务制度的适用，由国务院财政部门另行规定。

　　**第七十五条**　接受国家经常性资助的社会力量举办的人口和计划生育公益服务性组织和社会团体，依照本制度执行；其他社会力量举办的人口和计划生育公益服务性组织和社会团体，可以参照本制度执行。

　　**第七十六条**　下列人口和计划生育事业单位或者人口和计划生育事业单位的特定项目，执行企业财务制度或者同行业、相近行业财务制度，不执行本制度。
　　（一）纳入企业财务管理体系的人口和计划生育事业单位以及人口和计划生育事业单位附属独立核算的生产经营单位。
　　（二）人口和计划生育事业单位接受外单位要求投资回报的经营项目。
　　（三）经主管部门和财政部门批准的具备条件的其他人口和计划生育事业单位。

　　**第七十七条**　人口和计划生育部门所属的科学研究单位和学校，执行同行业事业单位财务制度。

　　**第七十八条**　省、自治区、直辖市财政部门与人口和计划生育主管部门，可以根据本制度，结合本地区实际情况，制定补充规定，报财政部、国家人口和计划生育委员会备案。
　　人口和计划生育事业单位应当按照本制度，根据单位实际情况，制定内部财务管理办法，并报主管部门备案。

　　**第七十九条**　本制度自2013年1月1日起施行。
　　附：

人口和计划生育事业单位财务分析指标

　　**一、**财务指标
　　1.预算收入和支出完成率，衡量人口和计划生育事业单位收入和支出总预算及分项预算完成的程度。计算公式为：
　　预算收入完成率＝年终执行数÷（年初预算数＋年中预算调整数）×100%。年终执行数不含上年结转和结余收入数
　　预算支出完成率＝年终执行数÷（年初预算数＋年中预算调整数）×100%。年终执行数不含上年结转和结余支出数
　　2.人员支出、公用支出占事业支出的比率，衡量人口和计划生育事业单位事业支出结构。计算公式为：
　　人员支出比率＝人员支出÷事业支出×100%
　　公用支出比率＝公用支出÷事业支出×100%
　　3.人均基本支出，衡量人口和计划生育事业单位按照实际在编人数平均的基本支出水平。计算公式为：
　　人均基本支出＝（基本支出-离退休人员支出）÷实际在编人数×100%
　　4.资产负债率，衡量人口和计划生育事业单位利用债权人提供资金开展业务活动的能力，以及反映债权人提供资金的安全保障程度。计算公式为：
　　资产负债率＝负债总额÷资产总额×100%
　　5.收入增长率，衡量人口和计划生育事业单位财政补助收入、事业收入、经营收入占全部收入的比重及增长幅度。计算公式为：财政补助收入占总收入的比率＝财政补助收入÷总收入×100%
　　事业收入占总收入的比率＝事业收入÷总收入×100%
　　经营收入占总收入的比率＝经营收入÷总收入×100%
　　财政补助收入增长率＝（当年财政补助收入÷上年财政补助收入－1）×100%
　　事业收入增长率＝（当年事业收入÷上年事业收入－1）×100%
　　经营收入增长率＝（当年经营收入÷上年经营收入－1）×100%

　　**二、**业务指标
　　1.宣传品进村入户率＝已发放宣传品的户数÷村总户数×100%。
　　2.避孕药具应用率＝已使用药具人数÷应使用药具人数×100%。
　　3.药具使用有效率＝使用药具有效人数÷已使用药具人数×100%。
　　4.免费孕前优生健康检查覆盖率＝完成免费孕前优生健康检查人数÷符合免费孕前优生健康检查条件人数×100%。
　　5．人口和计划生育培训人次：指人口和计划生育事业单位一定时期内（通常为一年）组织参加业务人员培训的人次。
　　6. 技术服务总量：指实施各类计划生育手术例数和生殖健康检查人次、育龄群众咨询指导人次以及育龄妇女随访服务人次。

© 北大法宝：（[www.pkulaw.com](https://www.pkulaw.com/)) 专业提供法律信息、法学知识和法律软件领域各类解决方案。北大法宝为您提供丰富的参考资料，正式引用法规条文时请与标准文本核对。
欢迎查看所有[产品和服务](https://www.pkulaw.com/helps/2.html)。法宝快讯：[如何快速找到您需要的检索结果？](https://www.pkulaw.com/helps/70.html)[法宝](https://www.pkulaw.com/helps/69.html)V6有何新特色？

扫描二维码阅读原文

**原文链接：**<https://www.pkulaw.com/chl/e2c7480f306b9d6abdfb.html>
